# A Plausible Association Between the Use of Elderberry and Autoimmune Hepatitis

**DOI:** 10.7759/cureus.24250

**Published:** 2022-04-18

**Authors:** Akshaya Ramachandran, Drashti Antala, Prasun Pudasainee, Sreelakshmi Panginikkod, Harsh Gupta

**Affiliations:** 1 Internal Medicine, AMITA Saint Francis Hospital, Evanston, USA; 2 Internal Medicine, AMITA Health Saint Francis Hospital, Evanston, USA; 3 Department of Rheumatology Allergy and Immunology, Tufts University School of Medicine, Boston, USA; 4 Gastroenterology, AMITA Health Saint Francis Hospital, Evanston, USA

**Keywords:** drug induced hepatitis, anti smooth muscle antibody, inflammatory liver disease, autoimmune hepatitis, elderberry

## Abstract

Hepatic injury due to dietary and herbal supplements can often share similar clinical characteristics with autoimmune hepatitis (AIH). *Sambucus *species, commonly known as elderberry, have been used in traditional medicine for centuries to prevent and treat respiratory problems. Although there are no clear reports on the association of elderberry with AIH or drug-induced hepatitis, there have been concerns about negative health manifestations linked to elderberry and the overproduction of inflammatory cytokines. In this article, we discuss a case of a patient who developed autoimmune hepatitis while on long-term elderberry-containing supplements and a probable association between the two.

## Introduction

Autoimmune hepatitis (AIH) is a chronic progressive inflammatory disorder commonly identified in females with a tendency to occur during the teen years and the fourth to sixth decades of life. Its distinguishing features include the presence of serum-specific autoantibodies and histopathology features of interface hepatitis [1-3]. Etiopathogenesis involves a multitude of independent factors such as human leukocyte antigen (HLA) allelic variants, infections, drugs, and the prevalence of autoimmune tautology, which cause an immunoregulatory imbalance, leading to a resultant T-cell mediated inflammation [1]. Steroids and immunosuppressants form the primary pillars of treatment, along with close monitoring of liver function tests to assess therapeutic response. The recommended duration of treatment is approximately two years, after which withdrawal can be considered. However, given the high risk of relapse, lifelong immunosuppressive therapy may be essential in most cases [3]. 

Natural supplements have been in use for decades in conjunction with modern medicine by mankind to treat a variety of common ailments. The rationale behind the use of such supplements is their presumed regulatory effect on the immune system, although studies conducted over the years have had mixed results [4-6]. One of the commonly used botanical supplements of interest with immunomodulatory effects is Sambucus nigra, also commonly known as elderberry, frequently used for the treatment of the common cold. The effects of elderberry on the production of pro-inflammatory cytokines such as IL-6, IL-8, and TNF have been controversial, with studies indicating a stimulatory versus inhibitory effect, leading to inconsistent interpretation [4]. We present a case of autoimmune hepatitis in a middle-aged female who was on long-term elderberry-containing supplements and developed autoimmune hepatitis. This case report aims to identify the risk factors associated with autoimmune hepatitis, debate the effects of elderberry on the immune system, and consider a causal association between the two entities.

## Case presentation

A 60-year-old Caucasian female with a medical history of Hashimoto thyroiditis and Medullary sponge kidneys presented to the hospital with nausea, decreased appetite, abdominal pain, and bloating for two days. She was on levothyroxine, indapamide, tamsulosin, potassium citrate supplements, and an over-the-counter elderberry supplement for several years. Her vital signs were stable. Physical examination findings were remarkable for scleral icterus, generalized jaundice, and abdominal distention without tenderness.

Initial laboratory studies revealed transaminitis with elevation of AST to 1821 IU/L (13-39 IU/L), ALT >2500 IU/L (7-52 IU/L), ALP of 232 IU/L (35-104 IU/L), total bilirubin of 10.5 mg/dL (0.0-1.0 mg/dL), and direct bilirubin of 5.66 mg/dL (0.00-0.20 mg/dL) (Table 1). Abdominal imaging studies, including ultrasound and CT scans, showed mild peri hepatic inflammation and a contracted gallbladder with no obvious stones or intrahepatic dilation. The acute hepatitis panel was negative; however, she was found to have a positive antinuclear antibody (ANA) screen and SSA (RO) Ab 1.4 (<1.0) (Table 1), which raised a concern for autoimmune hepatitis.

**Table 1:** Laboratory findings showing significant liver function and autoimmune workup AST: aspartate transaminase; ALT: alanine transaminase; ALP: alkaline phosphatase; SSA (RO) Ab: anti-Sjögren's-syndrome-related antigen A autoantibodies; SMA: smooth muscle antibody; ANA: antinuclear antibody

Laboratory findings	Results	Reference value
AST	1821 IU/L	13–39 IU/L
ALT	>2500 IU/L	7–52 IU/L
ALP	232 IU/L	35–104 IU/L
Total bilirubin	10.5 mg/dL	0.0–1.0 mg/dL
Direct bilirubin	5.66 mg/dL	0.00–0.20 mg/dL
SSA (RO) Ab	1.4 IU/mL	<1.0 IU/mL
SMA	1:160	<1:20
ANA screen	Positive	Negative

**Figure 1:**
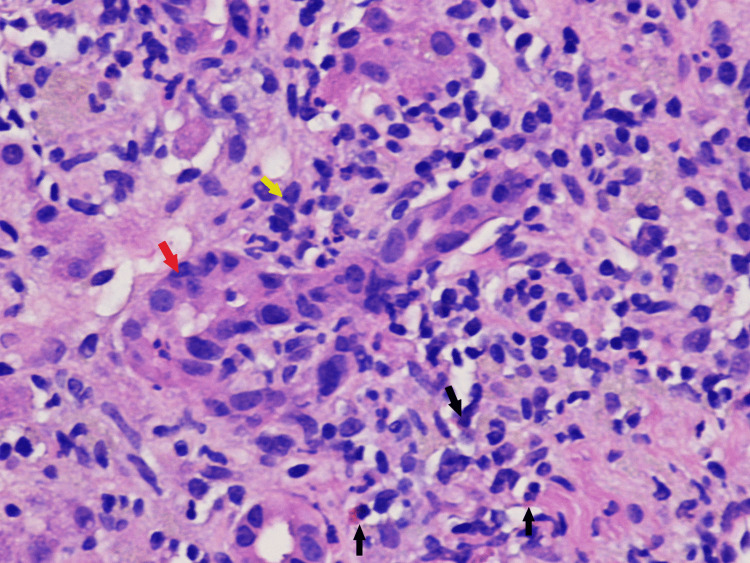
Histological finding of liver biopsy: bile duct epithelium and surroundings infiltrated with lymphocytes and sparse eosinophils H&E stain under light microscopy, original magnification 400×. Black arrows: eosinophils; red arrow: bile duct epithelium; yellow arrow: lymphocytes.

**Figure 2:**
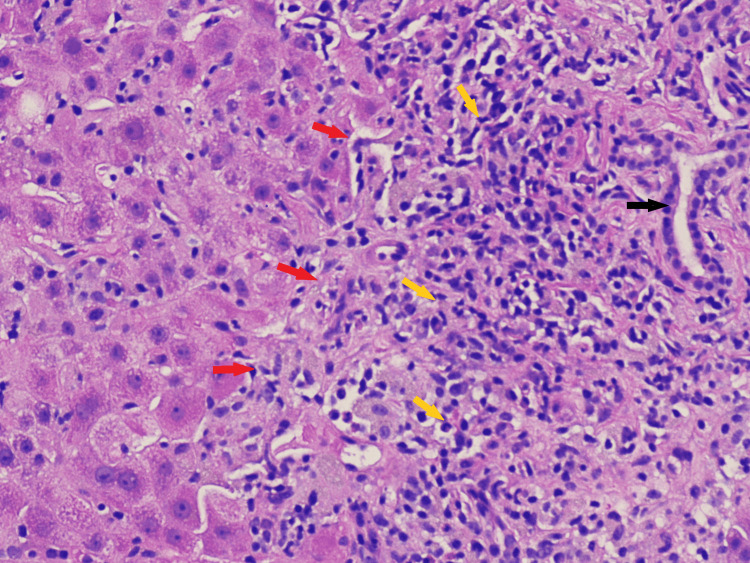
Interface hepatitis extending from a limiting plate consisting of an inflammatory infiltrate H&E stain under light microscopy, original magnification 200×. Red arrows: limiting plate; yellow arrows: inflammatory infiltrate of lymphocytes and plasma cells; Black arrow: bile duct.

Subsequent evaluation with a liver biopsy revealed extensive interface hepatitis, lymphohistiocytic infiltrate, eosinophils and neutrophils in the portal areas, periportal and lobular liver cell damage with piecemeal necrosis (Figure 1, 2).

Based on histological findings of the portal tract, the differential diagnoses included autoimmune hepatitis versus drug-induced hepatitis. She was instructed to stop taking natural supplements, and immunosuppressive therapy with daily Prednisone 40 mg was started for four weeks.

At a follow-up visit four weeks later, her repeat liver function tests improved to baseline. As elderberry supplements were presumed causes of hepatitis, they were not rechallenged. Prednisone therapy was tapered over two weeks, after which she was started on long-term maintenance therapy with azathioprine.

## Discussion

Autoimmune hepatitis is a chronic inflammatory liver disease depicted by a combination of elevated liver enzymes, the presence of specific serum autoantibodies and histological features of interface hepatitis, infiltration of lobules with lymphocytes, and plasma cells [3]. AIH is further classified into two types depending on the presence of autoantibodies: type 1 is positive for ANA and/or anti-smooth muscle antibodies (SMA), while type 2 is positive for liver-kidney-microsomal-type-1 (LKM-1) antibodies [2,3]. While studies have shown that it affects all ages, ethnicities, and genders, it has a bimodal distribution with peaks in the teens and the fourth to the sixth decade of life, with a female preponderance [1-3]. Literature suggests that the presence of one autoimmune disorder can increase the risk of an individual developing other autoimmune disorders [7]. One such commonly studied association with AIH is the presence of autoimmune thyroiditis [8,9]. In a case-control study, it was found that 30% of patients with AIH have concurrent extrahepatic autoimmune manifestations, out of which 51.4% were found to be diagnosed with autoimmune thyroid disease [9]. Another similar study revealed that autoimmune thyroid disease was the most common concurrent extrahepatic autoimmune disease with an 18% prevalence in those with autoimmune hepatitis [8]. On the other hand, in another cohort study, autoimmune hepatitis was found in 8.69% of patients with Hashimoto thyroiditis [10]. Uncertainty lies within the issue of whether autoimmune thyroid disorder is a risk factor or a cause of autoimmune liver disease, or vice versa, warranting further exploration.

The mechanism behind the development of autoimmune hepatitis is still not completely clear. A complex interplay between genetic predisposition, molecular mimicry, and effector-regulatory immunity is thought to be involved in the pathogenesis of autoimmune hepatitis. The presentation of an autoantigenic peptide to naïve CD4 T-helper cells (Th0) causes the secretion of proinflammatory cytokines like IL-12, IL-6, and TGF-B which leads to the development of Th1, Th2, and Th17 cells [11]. Th1 cells produce IL-2 and IFN-y, which activate cytotoxic T lymphocytes (CD8), thereby inducing the expression of HLA class I and II molecules on hepatocytes with further stimulation of macrophages. Th2 cells secrete IL-4, IL-10, and IL-14, leading to the maturation of B cells and plasma cells that produce autoantibodies [11]. Regulatory T cells (Treg) derived from Th0 cells are responsible for restricting hepatocellular injury by Th1, Th17, macrophages, complement, and natural killer cells [8,12]. Th17 cells produce cytokines that suppress Treg cells, thus amplifying liver damage along with the cascading effects of Th1 and Th2 cells, leading to the formation of characteristic lymphocytic and plasma cell infiltrates termed interface hepatitis [8].

As per another study, SPW 2, a neutral polysaccharide derived from Sambucus leaves, induced the secretion of IL-beta, IL-6, and TNF-alpha and increased the mRNA expression level of IL-6 and TNF-alpha in macrophages in vitro [6].

Sambucus species, commonly known as elderberry, is a traditional dietary supplement used to treat minor health conditions, particularly influenza [5]. While there are insufficient data to substantiate the effectiveness of elderberry, some studies have displayed its immunomodulatory effects. In a study done by Barak et al., Sambucol (elderberry) extract led to the production of inflammatory cytokines including IL-1 beta, TNF-alpha, IL-6, and IL-8 (2-45 fold) as compared to lipopolysaccharide, a known monocyte activator (3.6-10.7 fold) in vitro, thereby concluding that Sambucol extract activates the immune system by increasing inflammatory cytokine production [5]. Another comparable study revealed similar in vitro effects of Sambucus as well as its ability to stimulate the mRNA expression of inducible Nitric oxide synthase in macrophages, supporting the notion of elderberry’s potential regulatory effects on the immune system [6]. Considering the aforementioned discussion, it is worth debating whether elderberry may be contributing to the amplification of the cytokine production in vivo, inciting autoimmunity in genetically predisposed individuals - parallel to the development of autoimmune hepatitis in our patient with a history of a known autoimmune condition (Hashimoto's thyroiditis) on long-term elderberry supplements.

In addition to playing a vital role in the diagnosis, the purpose of a liver biopsy in AIH is to determine the prognosis, severity of the disease, and therapeutic response. The characteristic histological features of AIH are depicted by the presence of interface hepatitis or piecemeal necrosis, which is the inflammation of hepatocytes at the junction of the portal tract and hepatic parenchyma consisting of lymphocytes and plasma cells [13]. However, there exists approximately 25% overlap between AIH and acute viral hepatitis, with the degree of plasmacytosis being the discriminating factor, although 33% of patients with AIH do not exhibit plasma cells in the portal tract, and thus diagnosis should be made in the context of a clinical and serological background [13]. The presence of eosinophils seen in Figure 1 can correspond to a differential diagnosis of drug-induced hepatitis, although these findings can also be seen with AIH, making it difficult to differentiate between the two and establish a clear diagnosis. At the same time, our patient had a negative hepatitis serology, which helped in making the distinction. Another entity that is relevant to our case is a drug-induced liver injury with autoimmune features, which is a syndrome where one develops biochemical and histological features of autoimmune hepatitis after the ingestion of a drug or a herbal product [14,15]. It has a shorter incubation period with a gradual improvement observed after the inciting drug is stopped and can recur if it is re-introduced, whereas idiopathic AIH requires long-term immunosuppression [14,15]. In addition to the presence of a pre-existing autoimmune condition (Hashimoto’s thyroiditis) in our patient, there is a possibility that elderberry supplements could have initiated a self-propagating autoimmune process that could have caused the liver injury.

The cornerstone of treatment in AIH lies in limiting inflammation and preventing the progression to end-stage liver disease [11]. Debilitating symptoms like arthralgia and fatigue accompanied by hepatic inflammation, serum AST elevation ≥10-fold upper normal limit or AST elevation ≥5-fold upper normal limit concurrent with γ-globulin level ≥2-fold upper normal limit, and histological findings of bridging necrosis are absolute indications for treatment [16]. Treatment regimens include prednisone alone or in conjunction with azathioprine, which is continued until normalization of transaminases and IgG is achieved and is further maintained depending on the individual’s response and tolerance to therapy [2,11,16]. Considering the possibility of drug-induced hepatitis, our patient was instructed to stop the elderberry supplement and not to reuse it again. In addition, she was given immunosuppressive therapy to treat the significant hepatic inflammation as evidenced by severely elevated transaminases and liver biopsy. A four-week course of prednisone resulted in the resolution of symptoms and normalization of liver function tests. She was started on azathioprine maintenance therapy with a plan to potentially discontinue it after sustained remission for 18 months. Follow-up over time regarding the development of flare-ups, should they arise, will shed light on the subject of autoimmune hepatitis triggered by elderberry, as opposed to elderberry-induced hepatic injury. This could aid in recognizing the significance of using elderberry judiciously in patients with a history or predisposition to autoimmune disorders.

## Conclusions

Exposure to elderberry could be responsible for either the initiation or progression of autoimmune liver disease in the setting of genetic predisposition and molecular mimicry. Therefore, obtaining a meticulous history pertaining to medications is warranted, with an emphasis on over-the-counter supplements. A combination of serum-specific antibodies and distinct histological features in the liver biopsy can aid in formulating a diagnosis, while an initial course of steroids along with indefinite immunosuppressive therapy is essential to control inflammation as well as avert relapses.
